# Promoting Faculty Scholarship – An evaluation of a program for busy clinician-educators

**Published:** 2015-04-20

**Authors:** Stacia Reader, Alice Fornari, Sherenne Simon, Janet Townsend

**Affiliations:** 1 Department of Health, Physical Education and Wellness, Bronx Community College, Bronx, New York; 2 Hofstra North Shore-LIJ School of Medicine, Hempstead, New York; 3 March of Dimes - New Jersey Chapter, Sayreville, New Jersey; 4 Commonwealth Medical College, Scranton, Pennsylvania

## Abstract

**Background:**

Clinician educators face barriers to scholarship including lack of time, insufficient skills, and access to mentoring. An urban department of family medicine implemented a federally funded Scholars Program to increase the participants’ perceived confidence, knowledge and skills to conduct educational research.

**Method:**

A part-time faculty development model provided modest protected time for one year to busy clinician educators. Scholars focused on designing, implementing, and writing about a scholarly project. Scholars participated in skill seminars, cohort and individual meetings, an educational poster fair and an annual writing retreat with consultation from a visiting professor. We assessed the increases in the quantity and quality of peer reviewed education scholarship. Data included pre- and post-program self-assessed research skills and confidence and semi-structured interviews. Further, data were collected longitudinally through a survey conducted three years after program participation to assess continued involvement in educational scholarship, academic presentations and publications.

**Results:**

Ten scholars completed the program. Scholars reported that protected time, coaching by a coordinator, peer mentoring, engagement of project leaders, and involvement of a visiting professor increased confidence and ability to apply research skills. Participation resulted in academic presentations and publications and new educational leadership positions for several of the participants.

**Conclusions:**

A faculty scholars program emphasizing multi-level mentoring and focused protected time can result in increased confidence, skills and scholarly outcomes at modest cost.

## Introduction

In the context of shrinking hospital and federal support for education, academic departments and residency training programs must produce scholarship that critically evaluates and studies educational programs.^1^–^3^ Minimal dedicated time, competing demands, and limited skills or experience are challenges clinicians encounter in getting manuscripts developed and submitted.^4^ In addition, clinician educators face barriers to identifying and engaging mentors and typically experience less mentoring regarding academic career development than do basic science and physician scientist faculty.^5^–^7^ Both junior and experienced clinician educators, whose supported academic time is quite limited, represent a wealth of expertise and educational innovation that often does not get reported in the medical education literature.

A number of interventions promoting scholarship among clinician educators demonstrate modest but significant gains in faculty publications related to education. Typically, in the reported experience of authors cited in this paragraph, 30 to 50% of participants improve their publication record while greater than 50% present educational research or scholarship in peer reviewed settings. Peer writing groups are associated with increasing the number of presentations, peer-reviewed publications and collaborative projects.^8^–^12^ Faculty scholar programs provide a broad range of education competencies, including focused or integrated scholarship/medical education research that result in publications, participation in educational leadership, and increased career satisfaction for participants. Techniques to improve writing skills and productivity include feedback from senior advisors on writing skills; motivation to begin and assistance in sustaining writing projects; and demystification of the submission and publication processes. Additional techniques described in the literature are the incorporation of short writing periods during the workday; assistance meeting deadlines, reading and editing of work by peers or mentors; frequent meetings; forming a cadre of writing scholars; obtaining funding; protected time; finding additional venues for publication; and relationships with colleagues who publish.^13^–^27^

To address barriers to educational scholarship in the Department of Family and Social Medicine at the Albert Einstein College of Medicine and Montefiore Medical Center, education division directors, including a family physician (JT) and an educator (AF), sought and received federal funding to implement a faculty development program designed to support busy clinician educator faculty in conducting educational scholarship. Specific aims of the grant funded project were to: 1) increase department-wide faculty development activities in educational research and writing skills, and 2) increase the quantity and quality of peer-reviewed educational scholarship by departmental clinician educator faculty that addresses health disparities and Healthy People 2010 Objectives. This paper describes a Faculty Scholars program developed to meet the aims of the grant project, carried out between 2005 and 2009, reports on its successes and challenges, and provides recommendations for future research.

The program leadership team (JT, AF) designed an evaluation strategy that included qualitative and quantitative methods to answer the following questions:

1. Could a part-time faculty development program designed for clinician educator faculty with heavy patient care and teaching loads contribute to:
  - enhanced skills in educational scholarship and dissemination?
  - increased quantity and quality of peer reviewed publications of education research and innovations?
2. In what ways would the program elements contribute to participants’ perception of their confidence, knowledge and skills to disseminate educational scholarship as part of their career trajectory?

**Figure 1:**
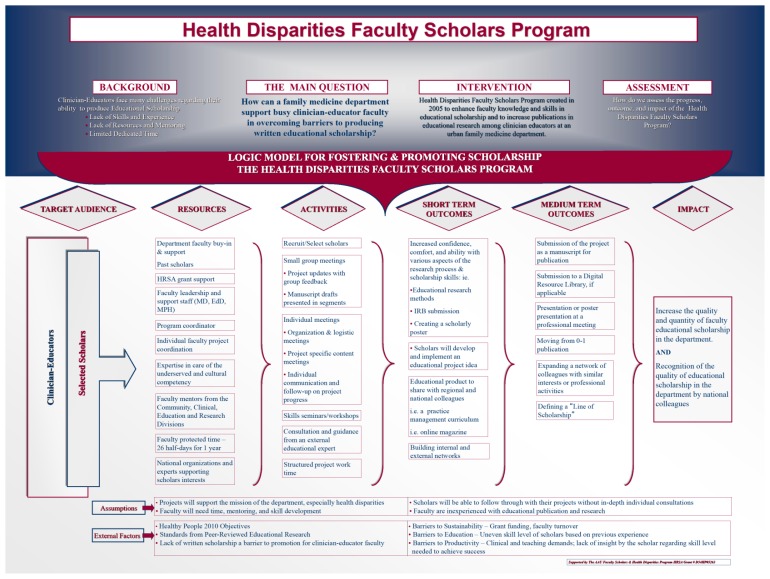
Logic model for fostering & promoting scholarship

The program leadership team (JT, AF) chose to use a logic model (Figure 1) to clearly outline the grant project and link specific aims, activities and outcomes. The theoretical and practical assumptions and principles of the program were also identified in the logic model framework, including the general inexperience of target faculty with medical education research, the likely heterogeneity of knowledge and skills at baseline, and the need for mentoring and involvement of departmental faculty members beyond the program leadership team (JT, AF). They decided the logic model would result in effective programming and offer greater learning opportunities for the program leadership, clearer and more accurate documentation of outcomes, and shared knowledge about what works and why in terms of achieving impact. We used this framework to track all program efforts and disseminate our outcomes at the local, regional and national level.^28^,^29^

## Methods

### Study design

Our design for this evaluative study of a faculty development intervention included quantitative and qualitative methods. We collected quantitative data through pre and post participation surveys of self-assessed skills with anchors that described the confidence level of participants as well as a follow-up questionnaire, conducted three years after program completion, about outcomes such as continued educational scholarship activity and dissemination of education scholarship. We conducted a qualitative assessment through semi-structured interviews regarding faculty scholar experiences including their self-perceived confidence, knowledge, and skills as well as reflections on their experiences and on strengths and weaknesses of the program. Our intent was to explore the change process for participating faculty. We chose these two types of data collection methods to have a richer data set and to assure that skills, knowledge and attitudes of participants were reflected in the data.

### Study sample and setting

Our study was carried out at the Department of Family and Social Medicine, Montefiore Medical Center/Albert Einstein College of Medicine in the Bronx, New York. The large, urban department’s responsibilities included medical student education, residency and fellowship training, clinical care, research and community service.

### Selection criteria

The program leadership team (JT, AF) recruited ten clinician educator scholars, based on a formal application and recommendations from supervisors, to participate in a part-time faculty development program with the goal of increasing educational scholarship skills and outcomes. The application process assessed the project plan, career goals, and scholarship development needs. Our selection criteria included relevance to the departmental mission of improving health in underserved communities and training physicians for practice in such communities.

### Human subject protection

As a structured faculty development program with necessary evaluation, our project met exempt criteria of the Institutional Review Boards of the medical college and teaching hospital. We confirmed the exempt status eligibility through inquiry to the IRB. We informed all participants in the program of the planned evaluation activities. During the application and orientation process, we informed each cohort about the evaluation plans, which included data collection and dissemination of results, and we reminded participants of the evaluation plan at the time of the follow up survey. In addition, participants gave implicit consent by completion of pre, post and follow-up surveys as well as participation in interviews. We deemed the risks for program participation to be minimal, with potentially significant benefits for career development. Because of the small size of the program and the need to understand the outcome for each scholar, which was an obligation of the funder for the principal investigators of the grant, we treated survey data and qualitative interviews confidentially. A trained staff person, who was not affiliated with the program or funded on the grant, conducted all qualitative interviews. We stored all follow-up data (tapes, transcripts, survey results) in secure, locked computers or cabinets, assuring privacy. Our selection process was equitable as all department faculty members were offered the opportunity to apply and applicants were supported in developing their application materials through meetings with grant faculty and review of draft materials.

### Intervention

We tested a part-time faculty development intervention, which is described as follows:

#### Structure

Our program was organized into three cohorts, with three application cycles, over the span of three years. This structure minimized the impact on clinical care at department practices and made best use of grant resources. Each cohort of 3–4 scholars actively participated for approximately 14 months. Each participant received protected time of 26 half-days for one year. Scholars received skills training through workshops and consultations with a visiting professor, the latter of which occurred both during and beyond their cohort cycle.

#### Activities

**Figure 2:**
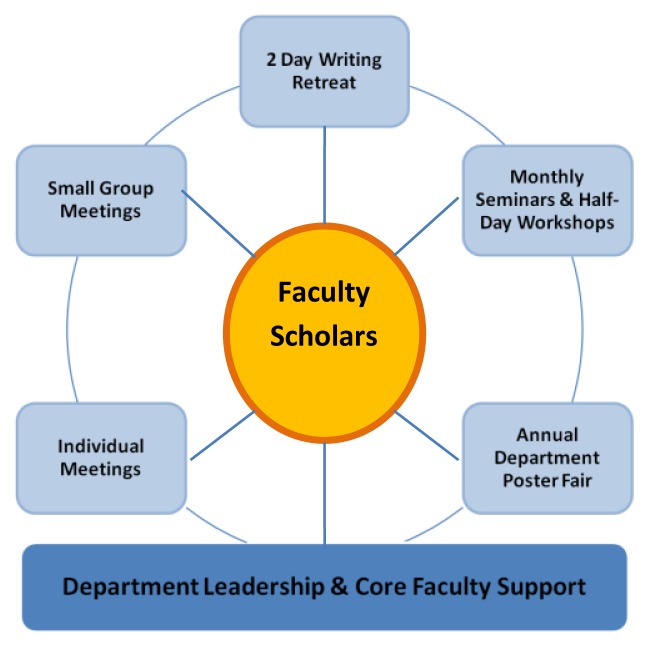
Support and organization/structure

The grant team (JT, AF, SS) included two principal investigators (a senior physician in the department and a doctoral trained educator) and a master’s trained project coordinator hired specifically to support both aims of the grant. The grant team reviewed the learning needs of the participants and considered their limited time availability. The grant team members therefore devised a multi-pronged approach to enhance the confidence, knowledge and skills of the participants and to achieve an outcome of increased educational scholarship. The grant team structured program activities in a part-time model that was feasible given the busy schedules of clinician educator participants and limited departmental resources (Figure 2). Scholars completed a self-assessment skill based questionnaire with a confidence scale as anchor descriptors, specific to their research skills, pre and post program to monitor their development over the program period. The grant team developed the questionnaire by reviewing the literature on approaches to enhancing research skills and success in clinical faculty, primarily based on the faculty needs assessment strategies used by Bland et al. at the University of Minnesota.^27^,^30^

Each cohort met monthly to discuss project plans, participate in project updates, present manuscript drafts, and to discuss challenges with projects. These meetings provided support, review and feedback from peers and faculty. The grant team designed monthly seminars and two half-day workshops to aid project development and writing; topics were tailored to address the immediate learning needs of each cohort. The master’s prepared coordinator (SS) met frequently with the scholars providing continuous project coaching and task management. She monitored their progress on project completion and provided support by using follow-up reminder emails that delineated next steps.

An annual two-day writing retreat focused on the scholars’ projects and dedicated project work time during which a visiting professor with expertise in educational research and scholarship development provided individual project consultation with each scholar. She also presented seminars on strategies for education project design, evaluation and writing for the scholars. For continuity, the same visiting professor returned each year. The visiting professor also gave Grand Rounds, other presentations and met with additional department faculty members during each annual visit, thus addressing the first grant aim of development of scholarship in the entire department.

An annual departmental Educational Scholarship Poster Session, introduced as part of this program, provided a capstone experience for the participants. Scholars designed and presented an academic poster on their individual projects. Poster design workshops were held beforehand to provide skills and content organization necessary for academic poster preparation.

Core project faculty met weekly to coordinate seminars and workshops, determine logistics, and develop strategies to assist in scholar learning and project needs. In addition, senior faculty members from the department’s Community, Clinical, Education and Research divisions provided periodic individual project consultation, scholar mentoring, and supported skill development through presentations to the group.

### Outcome measures

To inform program evaluation strategies, we created a logic model, previously described, to determine specific inputs and outputs of the Faculty Scholars Program (Figure 1), in an application similar to that of Armstrong and Barsion who used a logic model to frame a follow-up study of outcome of a faculty development program at the Harvard Macy Institute.^28^ We used educational activities and short-term and long-term outcomes to provide the framework for our quantitative and qualitative evaluation strategy using surveys and semi-structured interviews.

#### Scholars’ self rating of skill development before and after participation

The surveys included a self-assessment skills based questionnaire with anchors that describe confidence level of the participants, administered pre and immediately post participation in the program and a follow-up questionnaire, the administered 3–5 years after program completion to ascertain outcomes such as academic presentations, published scholarly products and continued application of skills in educational scholarship. Formative evaluation took place during the program in the form of informal scholar feedback, which assessed the content, presentations, and applicability of the seminars, workshops, and retreats.

#### Educational themes

We framed the interview guide for the qualitative semi-structured interviews from short- and medium-term outcomes of the program logic model, employing primarily open-ended questions with prompts. A trained research assistant conducted the interviews with each scholar, who had no affiliation with the scholars program, within one year of program completion. The interviews lasted between 30 and 60 minutes. Interviews for Cohorts 1 and 2 were conducted in person, tape recorded and transcribed verbatim. Interviews for Cohort 3 were conducted by phone with detailed field notes recorded.

### Data analysis

We used descriptive statistics to summarize our findings. We found that the small sample size (*n* = 10) was not sufficient to provide reliable inferential analyses of change over time, so descriptive summaries were generated to examine improvement in self-reported ability in selected skill areas. Summaries included frequencies and percentages of responses in each category prior to intervention (Unable to do, Can do with help, Can do independently). Improvement in skills areas was summarized in two ways: as the percentage of scholars who could not do the selected skill independently at pre-test, but showed any improvement (could do skill with help, or independently) at post-test, and also as the percentage of all respondents who could do the skill independently prior to and following the intervention. We generated summaries using SPSS (IBM Corp. Released 2013. IBM SPSS Statistics for Windows, Version 22.0. Armonk, NY: IBM Corp.).

Core faculty for the program, all of whom had graduate level or faculty development training in qualitative methods, independently reviewed the interview transcripts or field notes and identified themes using independent open and axial coding. We did not use qualitative analysis software. After independent coding, we held consensus meetings among the three reviewers to compare emergent themes and reach consensus. Next, the three reviewers collapsed the structured fellowship themes into larger categories. Finally, a fourth reviewer (SR), an educator who had not participated in the program, reviewed the transcripts and field notes, categorized themes, and concurred with the group’s findings.

## Results

### Scholar demographics

The scholars (eight women and two men) ranged in age from 32 to 54 years (mean 45) and described themselves as novice researchers (Table 1). Eight professors and one was an instructor, and one, a new residency program behavioral science faculty member employed by the hospital partner, did not yet have an academic appointment. Participants had an average of 14 years of experience teaching both medical students and residents and 16 years in clinical practice. 90% identified their primary professional role as clinician educators. Though full time employees of the teaching hospital, all participants had heavy patient care and clinical supervision roles in the residency program, thus their academic involvement was “part-time” and they had not previously had protected time for scholarship.

#### Scholars’ self rating of skill development before and after participation

Scholars’ self-reported ability in research skills areas are shown in Table 2. At baseline, for all skills, a majority of scholars reported being unable or requiring help to complete the skill (range: 50% to 90%). Self-reported ability improved for all skills among those reporting that they were unable to complete the skill independently prior to the intervention. Scholars’ ratings improved most for the skills of curriculum design, building internal and external networks and creating a scholastic poster, with more than 80% of those unable to complete the skill independently prior to the intervention showing improvement. Further, the percentage of scholars that could perform skills independently increased for all skills except choosing a quantitative method (Table 3). In addition, the short and medium term outcomes specified in the logic model (Figure 1) were achieved; the scholars themselves attributed these improvements directly to participation in the scholars program.

#### Educational themes

We conducted semi-structured interviews with all ten scholars after participation in the program. The emergent themes we found and exemplars are listed in Appendix 1. All scholars reported four key structural factors that facilitated progress. First, a protected block of time every other week, away from patient care and teaching responsibilities, allowed for exclusive focus on their projects. Second, a dedicated master’s prepared coordinator coached the scholars on tasks including project management, editing, feedback, and setting deadlines. Third, engagement of dedicated project leaders provided direction, support and clarification on project issues.

Finally, the scholars reported that the visiting professor lent external validation to their work. The scholars utilized sessions with the visiting professor for various purposes such as obtaining feedback, discussing professional aspirations and learning about current national research priorities and funding sources.

Scholars noted that the project leadership and research faculty mentors provided strong mentorship. As one scholar mentioned,

“They provided moral support, which is actually very important to me when I am engaged in something that I really don’t have a lot of experience with.”

Another stated that mentorship “…helped me focus my energies and produce what I need to produce.”

We found several other factors were important to the scholars’ success. For example, most scholars viewed the program’s didactic sessions as helpful for completing their current project and acquiring a skill set applicable to future academic scholarship. The most useful presentations focused on conducting a literature review, designing survey methodology and managing references. The value of peer interaction emerged as another consistent theme. Many of the scholars found that participating as a cohort provided structure, concrete and moral support, and confidence in their work. Some scholars noted that working collaboratively with peers expanded their perception of educational scholarship.

One scholar reported that,

“Before I became a scholar, I had tunnel vision and really had not thought seriously about getting involved, collaborating with others around educational scholarship. This really enlightened me, brightened my future, allowed me to make connections that I needed and more importantly, it made me self-reflect.”

Peer review of work also allowed the scholars to learn about barriers encountered and skills needed for the projects of other cohort members. Scholars found that continuous redrafting of manuscripts for peers provided useful and comprehensive feedback on the writing process.

Another scholar’s reflection supports these findings from the interviews:

“The drafting portion [of the group meetings] for the manuscript …was really useful because that makes it a step by step process instead of a big intimidating thing. And then peer and faculty feedback just makes you … go through the stages of drafting and redrafting …much more quickly because of second eyes-sees much more easily what makes no sense at all.”

After completing the fellowship, participants reported increased comfort, confidence and competence in conducting scholarly work, as noted in Table 2. Scholars also reported more career satisfaction and inspiration to pursue academic career goals.

#### Organizational Challenges

One significant challenge with program implementation identified by the scholars included difficulty in obtaining “true” protected time. Several scholars noted competing priorities especially lack of coverage when away from clinic.

As one stated,

“The expectation is that even though you are not at the health center, you’re still covering your patients so you are still liable to get phone calls, see patients in the hospital…”

Other scholars reported difficulty scheduling protected time because administrative staff was not always supportive of the project. In addition, some participants noted initial difficulty transitioning between clinic and academic work, but over time learned to manage the transition better. Some scholars noted a decrease in the frequency of monthly small group meetings. This occurred due to competing schedule demands for participants, especially in finding a common time to meet. Scholars in one cohort noted a need for more efficient and effective meetings.

As one scholar reported,

“I didn’t feel the time was always well used…it might have been useful to have shorter time per project…and to focus the discussion on very real practical issues we were all facing.”

#### Program Structure and Sustainability

Scholars suggested several approaches for the department to encourage faculty to engage in more scholarly work. Scholars felt it was imperative to have protected time to engage in scholarly activities, gain the skills needed to write grants and eventually secure funding for educational projects. Others suggested that encouraging ongoing collaboration within the department was absolutely necessary for career development. In addition, most scholars suggested the department fund both a dedicated staff person to assist with logistics and editing and mentoring time for the research faculty.

#### Impact of Program

Nine of 10 scholars responded to the follow-up questionnaire, conducted three years after program completion. Publications and presentations resulting from the faculty scholars’ projects are presented in Table 5. At the time of the follow-up questionnaire, scholars had published five peer reviewed articles related to their projects. Three of the nine scholars continued work related to their scholar’s project. Two scholars had manuscripts in preparation, based on their project. Eight scholars (89%) are currently working on new research and/or scholarly projects. In the follow-up survey, scholars identified the skills developed during the grant funded initiative that they are applying to new scholarly projects. (Table 6) The participants are involved in a variety of new scholarly efforts, including a feasibility study regarding misoprostol; innovative resident selection interviews; teaching about uncertainty in medical decision making; interprofessional training for the patient-centered medical home; and benefits of reflective writing in medical education.

In the follow-up survey all respondents reported benefitting from faculty and staff mentoring as well as from peer coaching and felt they gained positive momentum from departmental recognition of their work. All reported increased confidence in planning scholarly projects. Over half reported increased project management skills and a familiarity with qualitative and quantitative data analysis preparation of an academic poster, and preparation of a manuscript. In reflecting on the impact of the program, many of the participants felt the scholars program created a strong sense of collaboration and creativity. For example, one scholar stated,

“One of the benefits of the … faculty scholars’ project is that it created a community of support for a variety of projects that faculty members felt passionately about. In doing so, it energized people to work on and complete projects. It also gave people ideas and stimulated creative approaches to their projects. It also fostered collegiality and a sense of common purpose, a healthy antidote to the isolation generated while working on scholarly endeavors”.

#### Other related outcomes

One participant became the director of the department’s federally funded primary care faculty development program subsequent to her participation in the program. Another accepted a faculty leadership role in a new family medicine residency program in the region. The on-line journal initiated by another scholar has been very successful, offering a weekly venue for other educators and clinicians to publish peer reviewed reflections and led to two books of collected essays and poems from the journal. Another participant was recruited to be part of the core residency faculty in the department.

## Discussion

Our findings are consistent with previous reports in the literature, which found that scholars’ programs that include protected time, mentoring, and active participation in a peer group support lead to progress in written scholarship and enhanced confidence and identity as scholars.^31^

New contributions to the literature regarding such faculty development interventions reported in our study include the use of a consistent visiting professor over time, the integration of a master’s prepared coach who worked individually with faculty, and a program structure involving only modest protected time. In the following sections we will discuss the program elements associated with the achievement of short and medium term outcomes that we had delineated in the logic model, including increased confidence and skills, implementation of an educational scholarly project, building of internal and external professional networks, academic presentations and publications, and defining a line of scholarship.

### Importance of mentoring

Scholars benefitted from multi-level mentoring and coaching from the project coordinator, project leadership, an external expert (a visiting professor), department research faculty, and peers. The role of the dedicated master’s prepared coordinator, who devoted fifty percent full time effort, proved to be invaluable. She provided individual task-focused coaching that reduced scholars’ projects down into small, discrete steps, allowed scholars to gain confidence and move forward. The guidance and follow-up on tasks provided through the coaching supports the findings of others regarding the necessity for a structure that supports accountability.^16^,^17^,^24^ Leadership offered overall direction for the scholars’ projects, helped clarify their thinking, and brokered additional support and resources from the department.

Engaging a single visiting professor over the course of the program resulted in consistency and continuity of input. At each visit, the visiting professor’s individual consultations, feedback on projects and career goals, group meetings and formal presentations/workshops on research skills provided a sense of external validity to the scholar’s work, which inspired the scholars to continue their projects, despite competing demands.

The didactic sessions leveraged departmental expertise of senior faculty members and were deemed crucial by the scholars as they increased scholars’ research and scholarly skills and provided confidence in their ability to complete their current projects and future academic work.

### Cohort Effect

Our study confirms the findings of others regarding the power of peer mentoring.^8^–^12^,^29^,^31^ Participating as a cohort was important because being members of a group that received coaching and peer interaction was highly valued. Our scholars’ experience mirrored that of other similar programs in creating a community of practice^32^ with common concerns, shared values, and mutual respect without the barriers of hierarchy. As reported in other studies, our study found that regular peer meetings validated the scholars’ interest in educational issues, allowing for more personal feedback, dialogue, safety, and an opportunity to practice new skills and to solve problems. This process also contributed to scholars’ motivation, confidence and excitement about engaging in scholarship. Continuous redrafting of manuscripts provided insight and stepwise feedback on the writing process. Our structure demonstrated a feasible scholarship development model with individual and group components that addressed a diverse group of learners that otherwise might never have engaged in educational scholarship.

Our experience regarding the value of mentoring from different individuals with various types of expertise and approaches to mentoring, including senior faculty, program staff and peers, is consistent with recent findings reported in the literature regarding the impact of networks of mentors and “horizontal mentoring” by peers.^30^ In fact, a qualitative study of former recipients of NIH mentored career development awards demonstrated the unlikelihood that a single mentor can meet all needs, the importance of mentor networks, and the value of peers as mentors, particularly in regard to pooling resources and mutual learning.^33^ This supports our finding of the power of multiple mentors and peer cohorts. The program leadership and staff, as well as the visiting professor, found it very gratifying to see the growth in skills, confidence and commitment to scholarship among most of the participants, who previously had not experienced support in evaluating and writing about their contributions to education in the department.

### Challenges

Funding allocations over 3 years limited the project to ten scholars, and thus, the impact of the program in the department. The diversity of scholars ready for scholarship development and their varying schedules made it difficult to plan content sessions and meetings, and thus to deliver a consistent set of learning activities to each scholar. As anticipated, participants reported the need for protected time to conduct educational research. Although the allotted time was modest, the scholars agreed it was essential to allow them to focus on their scholarship and achieve consistent progress. The lack of long term protected time past twelve months limited their ability to progress in their projects in an efficient way and therefore had an impact on definitive outcomes within the 3 years of grant funding. We were not surprised that scholars found their protected time frequently interrupted by clinical and teaching responsibilities, given the patient care and clinical teaching commitments of the sponsoring department and the multiple professional duties. This issue required early and repeated attention by program leadership to communicate and collaborate with clinical administrative staff to assure scheduling of protected time. In addition, several scholars discussed a preference for one specific mentor assigned to their entire project instead of mentoring by various program faculty and peers. Due to limited faculty resources the design of the program assured the workload of longitudinal mentoring was shared by a few expert faculty and the scholars’ peer group. Though several research faculty members volunteered their time by providing guidance to the scholars, scholars reported some difficulty in obtaining consistent assistance on research design and methods from these faculty members, due to their competing obligations.

### Limitations

Our study has several limitations. The scholars selected for the program came from a defined pool of clinician educators in one clinical department, which has a focus on delivery of primary care in an urban underserved ambulatory setting and a parallel focus for residency/fellowship training, reducing the generalizability of our findings. Only 9 of 10 scholars responded to the 3-year follow-up survey. The data reported by the scholars of their pre and post confidence with research skills during the program and the additional data collected in 3 year follow-up surveys were both self-reported and may have reflect scholars’ omission of information or attributing the results to events that occurred outside of the funded fellowship. Several of the scholars projects had goals and outcomes that diverged somewhat from the intended outcomes of the program, such as creating curriculum and developing an on-line reflective journal, all relevant to the Department mission but less aligned with traditional mechanisms of publishing educational research; therefore some scholars were less focused on achieving a traditional peer reviewed publication as an outcome measure and resulted in projects that fulfilled a boarder definition of scholarship. This created some ambiguity about whether program objectives were met by these participants. The intended focus on health disparities was not fully achieved in that not all projects directly addressed the topic. The participants’ strong interest in their topic and ownership of their project proved most important in assuring progress and completion. A final limitation was a self-report survey to determine if the Scholars continued their research efforts post grant completion. This was the most efficient way to collect current data from the Scholars, as their CVs may not be current with their ongoing research efforts.

## Conclusions

Our experience demonstrates both the feasibility of and successes associated with a part-time scholars program in medical education research for teaching faculty as an alternative to a full fellowship that includes significantly more protected time and resources.

Faculty members who previously had not engaged in medical education research and written scholarship represent an untapped resource of experience and perspective. Our experience demonstrates that it is possible to support scholarship by novice clinician educators, resulting in meaningful engagement, acquisition of relevant knowledge and skills, and measurable progress in producing scholarship. Engagement of a consistent external consultant and a dedicated master’s level coach added value and is relevant to departments wishing to embark on a similar endeavor. Our findings suggest that future programs may confer additional benefit if they provided a longer period of protected time and greater funding for scholars. It is essential for leaders of such programs to be proactive in scheduling true protected time for faculty to avoid distractions from competing demands and priorities. The importance of these infrastructure and support mechanisms was highlighted in the report by Simpson et al.^34^ of the 2006 Consensus Conference on Educational Scholarship convened by the Association of American Medical Colleges (AAMC) Group on Education Affairs (GEA) to outline a set of documentation standards and infrastructure needs for use by educators, academic promotion committees, and leaders in academic medicine.

Future research in this area might explore the use of technology, such as webinars with linked resources, to enable clinicians to participate from various clinical or teaching sites. Researching in more depth the experience and evolving identities of mid-career teaching faculty who embark on medical education scholarship might identify success factors and ways to address barriers. Testing the impact of implementing the unique components of our program, with cost/benefit analysis, might demonstrate the value of further iterations of our approach in this era of limited resources. Comparing the value of a single mentor who brokers input from other faculty members to a multi-mentor approach such as ours would be valuable, given the feedback from our scholars. O’Sullivan and Irby’s call for an expanded model of research^35^ on faculty development that focuses on two communities of practice, that of the participants and faculty engaged in a faculty development program and that of the workplace teaching practice where faculty members live and teach offers a framework for exploring more integrated models of faculty development.

## Figures and Tables

**Table 1:** Description of scholars and their projects (*N*=10)

Cohort 1	Project Description
Family MD, mid-career	Development and Assessment of a Residency Curriculum on Practice Management in Community Health Centers
Family MD, mid-career	Development of an On-line Reflective Journal
Family MD. MPH, early career	Impact of Abortion Training on Family Medicine Residents’ Pregnancy Options Counseling Skills
Family MD, mid-career	Development of Teaching Strategies for Conducting Culturally Sensitive Family Meetings at the End of Life

**Cohort 2**	

Family MD, mid-career	Survey of Family Medicine Residents and Program Regarding Interest in Family Medicine Obstetrics Fellowship and revision of previously drafted Review of the Women’s Health Content on Family Medicine In-training exams
Family MD, mid-career	Use of Reflective Learning Exercise in a School Health Rotation for Assessment of Learning
Family MD, mid-career	Qualitative Study of Resident and Faculty Comfort with Uncertainty in Clinical Decision Making

**Cohort 3**	

Family MD, early career	Care Based Teaching in Clinical Wound Healing
PhD Psychologist, early career	Rapid Assessment of Mental Health Need in Urban Primary Care
Family MD, MPH, early career	Making Intrauterine Contraception Available for Adolescents: Where are the Pediatricians?

**Table 2:** Pre-intervention skills ratings and improvement in 2009

	Prior to intervention						Following intervention	
	Unable to do		Can do with help		Can do independently		Any improvement*	
Skill	n	%	n	%	N	%	n	%
Literature review	-		5	55.6	4	44.4	2	40.0
Proposing project	-		7	77.8	2	22.2	3	42.9
Defining scope of project	-		10	100.0	-		3	30.0
Needs assessment	4	40.0	4	40.0	2	20.0	6	75.0
Project design	2	20.0	8	80.0	-		5	50.0
Developing methodology	3	30.0	6	60.0	1	10.0	4	44.4
Choosing method- quantitative	4	40.0	5	50.0	1	10.0	3	33.3
Choosing method- qualitative	6	60.0	3	30.0	1	10.0	5	55.6
Curriculum design	1	10.0	4	40.0	5	50.0	4	80.0
Building internal network	2	20.0	7	70.0	1	10.0	8	88.9
Building external network	5	50.0	5	50.0	-		8	80.0
IRB submission tasks	2	20.0	4	40.0	4	40.0	2	33.3
Implementing a project	-		9	90.0	1	10.0	2	22.2
Designing evaluation plan	6	60.0	2	20.0	2	20.0	6	75.0
Analyzing/interpreting outcomes	5	50.0	4	40.0	1	10.0	7	77.8
Selecting venue for dissemination	3	30.0	6	60.0	1	10.0	6	66.7
Sharing product regionally/nationally	-		6	60.0	4	40.0	4	66.7
Organizing scholastic poster	1	10.0	6	60.0	3	30.0	4	57.1
Creating scholastic poster	2	20.0	5	50.0	3	30.0	6	85.7
Outlining manuscript	3	30.0	5	50.0	2	20.0	5	62.5
Writing intro section	-		8	80.0	2	20.0	4	50.0
Writing methods section	2	20.0	6	60.0	2	20.0	3	37.5
Writing results section	2	20.0	7	70.0	1	10.0	4	44.4
Writing conclusion section	2	20.0	7	70.0	1	10.0	5	55.6
Submitting completed manuscript	3	30.0	5	50.0	2	20.0	5	62.5
Defining professional line of scholarship	2	20.0	7	70.0	1	10.0	4	44.4

* Denominator is number of scholars unable to complete skill independently prior to intervention

**Table 3:** Scholars’ ratings of independence in research skills in 2009

Skill	Prior to intervention		Following intervention	
	*n*	%	*n*	%
Literature review	4	44.4	6	66.7
Proposing project	2	22.2	5	55.6
Defining scope of project	-		3	30.0
Needs assessment	2	20.0	5	50.0
Project design	-		3	30.0
Developing methodology	1	10.0	2	20.0
Choosing method- quantitative	1	10.0	1	10.0
Choosing method- qualitative	1	10.0	2	20.0
Curriculum design	5	50.0	7	70.0
Building internal network	1	10.0	7	70.0
Building external network	-		5	50.0
IRB submission tasks	4	40.0	6	60.0
Implementing a project	1	10.0	3	30.0
Designing evaluation plan	2	20.0	3	30.0
Analyzing/interpreting outcomes	1	10.0	3	30.0
Selecting venue for dissemination	1	10.0	4	40.0
Sharing product regionally/nationally	4	40.0	8	80.0
Organizing scholastic poster	3	30.0	7	70.0
Creating scholastic poster	3	30.0	8	80.0
Outlining manuscript	2	20.0	5	50.0
Writing intro section	2	20.0	6	60.0
Writing methods section	2	20.0	4	40.0
Writing results section	1	10.0	4	40.0
Writing conclusion section	1	10.0	4	40.0
Submitting completed manuscript	2	20.0	5	50.0
Defining professional line of scholarship	1	10.0	4	40.0

**Table 4:** Table 5. Scholars’ project related presentations and publications (*N*=9)

*Publications/Presentations as of October 2012*	*N*
Oral presentation at a national or regional conference	6
Poster at a national or regional conference	6
Peer Reviewed Book Chapter	2
Peer Reviewed Journal	2
Peer Reviewed Online Resource Library (such as FMDRL and MedPortal)	1
Other forms of scholarship*	1
None	2

* One scholar developed a new on-line magazine for reflections on health care from patients and health professionals (http://pulsemagazine.org/index.cfm).

**Table 5:** Table 6. Scholar self-report of skills used in their current work as of 2012 (3–5 years post baseline) (*N*=9)

Skill Set	*N*	% of scholars using skills in current work
Academic poster Preparation	8	88%
Literature review	9	100%
Preparation of a manuscript	7	75%
Qualitative study design	5	56%
Qualitative data analysis	6	63%
Quantitative data analysis	3	33%
Survey methodology	3	33%
None	0	0%

**Appendix 1:** Themes Reported by Scholars and Supporting Quotes (*N*=10)

Theme	% of scholars who commented on theme	Quotes Supporting Theme
Protected time	100	“Really what I needed was dedicated time so I’d have relief time from clinic to work on the project…it gave me a chance to… really move the project forward a lot more than I would have without it.” “Having a block of protected time was tremendously helpful. Just …being able to conceptualize what I was doing as opposed to doing little steps-to see the whole process.” “That six months of time, of one-half day a week was huge for me. I needed that kind of time carved out, set aside time to get started [on my project].” “Having the protected time…I’d never had protected time in a block, and I was able to draft a paper, which I was just amazed. I used to have so much trouble pulling my thoughts together and not a lot of time to see the whole picture at once. And having that protected time just allowed me to progress.”

Coordinator support	100	“The masters prepared person, who really has a significant amount of her time dedicated to helping us with our projects…has been essential in terms of helping to set goals, deadlines and plans. And she was directly useful in project design and editing.” “The coordinator was an absolute incredible value to this faculty development and to the overall aid of the project” “I think the coordinator has helped me a lot in terms of data analysis of my project.” “The coordinator was very excited about our projects….and maintained a consistent level of interest”. “The coordinator had very clear thinking, was able to help me move ahead, push me but not too hard, easy to work with and very organized, she was just a huge help in moving me forward…”

Dedicated leadership	100	“They were really helpful with direction, logistical problems, helping to clarify thinking, and ready to review things…over time, they provided just the right amount of support as I went along and were very open in terms of getting me funding.” “They provided moral support, which is actually very important to me when I am engaged in something that I really don’t have a lot of experience with.”

Visiting professors	100	“Having her there, getting her perspective and feedback was terrific….it was helpful to get someone from the outside and realize…other people are doing all kinds of neat stuff and that is very inspiring. I think that is of great benefit to us.’ “The individual sessions were crucial…I used them in different ways. The first one I used for my project… the next one I used for my professional aspirations. It was very helpful to have someone outside the department listen to where we were going and what we had to say’ “I think some sort of external validation happens when there’s a visiting professor. It provides some validation that what you are doing is worthwhile and its working out and so forth. “The visiting professor had tremendous energy, enthusiasm and interest. And she is really an expert in producing papers and doing research…this was incredible helpful.”
Comfort, confidence, & competence in scholarly work	100	“I had not had a lot of exposure to academic undertakings and in giving me this exposure it made me realize that this [academic work] is something I could do, even if I didn’t have all the skills I could learn the skills, and I could acquire what it would take to do it.” “It [the scholar’s project] did increase self esteem and confidence and it sparked and interest in me doing, continuing this type of work.” “I learned that I could write. I always knew that but now I really know I can write.” “I felt pretty confident….when I was able to present it as a poster at a national conference.”

Didactic sessions	70	“The workshops were important. I did not end up using the survey writing. I have not done the abstract yet. But you know, all those things were really important. Those were the kind of skills, and even though some of the skills were more in quantitative research that I’m not using right now, it was just very helpful to have those set out… crucial.’ “There were sessions that were offered on specific topics that were very helpful. There was a session on survey methodology that was very useful. A session on managing your research sources that type of thing. Those sessions were very helpful” “I think really the most helpful was the literature review. The literature session and learning to use endnote web and developing a resource listing was very helpful.”

Small group/peer review of work	70	“Hearing about other people’s projects was a strength…people had different projects and although they might not be directly related to my own projects but I was learning about other people’s projects and some of the skills that they were acquiring or some of the barriers they were facing, and how they were overcoming them.” “Having peer review of your work and suggestions were really great and that is something that I would hope to continue in some way in our department.” “Project updates were helpful because it kept you moving along on what you were doing. The peer review of writing was very, very helpful.” “When they [the small group] happened I thought they were really useful …there were three in our cohort and with one of the cohort, we are co-investigators for each others projects, which is really wonderful. Anther cohort member and I are talking about starting a project together in the fall so I feel like that was extremely fruitful as well as just the feedback and interactions being fruitful, you know, it produced more than that.” “The drafting portion [of the group meetings] for the manuscript I felt was really useful because that makes it a step by step process instead of a big intimidating thing. And then peer and faculty feedback just makes you much further through, more quickly makes you go though the stages of drafting and redrafting I think much more quickly because of second eyes-sees much more easily what makes no sense at all.”

Cohort concept	60	“I really benefit from having someone help me to structure so that I can move forward quickly. And it really helps me to have to have a group to both push me forward and offer concrete support, but there is also a real moral support to it to sort of lend confidence that it’s worth all of this time and effort. So I felt working in a group was exponentially better for me than working by myself.” “Working collaboratively, working with projects, working in small groups, is absolutely necessary in this type of learning environment.” “There is always time to appreciate other people at different levels of growth. You always learn from what everyone else is doing.” “Before I became an AAU scholar, I think I had tunnel vision and really had not thought seriously about getting involved, collaborating with others around educational scholarship. This really enlightened my, brightened my future, allowed me to make connections that I needed and actually, more importantly, really made me self-reflect that I have a lot more capabilities than just taking care of patients and teaching residents. I really have an art that actually needs to be expanded on, and that’s the art of writing…” “The other faculty that were in the cohort with me were crucial…because it was people to work with. It keeps you going. They get kind of excited about your idea. They’re supportive. They’re interested in hearing what’s going on. That also was really important. And then within our group, I and [another member] were able to work on each other’s projects. So that, again, is support.”

Role of scholarship in career satisfaction	60	“It made me think about where I want to be in five or ten years from now and where do I want to be, what do I want to be doing, and how can I get there? What skills do I need to get there? It’s causing me to pause, and reflect, and see what I am doing now and am I in a position right now that is going to promote or encourage my professional development and growth?” “I think it improved my current satisfaction with my career from the standpoint of especially getting to know people a little more and how things work in the department.” “It’s a big notch up because I did something I have been wanting to do…even if I never publish anything, it [scholarship] enriches my practice and it really informs my teaching.” “It [scholarship] is having a growing value to me and its an area I would like to grow into more.”
